# Rationalising the role of Keratin 9 as a biomarker for Alzheimer’s disease

**DOI:** 10.1038/srep22962

**Published:** 2016-03-14

**Authors:** Joanna L. Richens, Hannah L. Spencer, Molly Butler, Fiona Cantlay, Kelly-Ann Vere, Nin Bajaj, Kevin Morgan, Paul O’Shea

**Affiliations:** 1 Cell Biophysics Group, School of Life Sciences, University of Nottingham, University Park, Nottingham, United Kingdom; 2 Department of Neurology, Nottingham University Hospitals NHS Trust, Queen’s Medical Centre, Nottingham, United Kingdom; 3 School of Life Sciences, University of Nottingham, Queen’s Medical Centre, Nottingham, United Kingdom

## Abstract

Keratin 9 was recently identified as an important component of a biomarker panel which demonstrated a high diagnostic accuracy (87%) for Alzheimer’s disease (AD). Understanding how a protein which is predominantly expressed in palmoplantar epidermis is implicated in AD may shed new light on the mechanisms underlying the disease. Here we use immunoassays to examine blood plasma expression patterns of Keratin 9 and its relationship to other AD-associated proteins. We correlate this with the use of an *in silico* analysis tool VisANT to elucidate possible pathways through which the involvement of Keratin 9 may take place. We identify possible links with Dickkopf-1, a negative regulator of the wnt pathway, and propose that the abnormal expression of Keratin 9 in AD blood and cerebrospinal fluid may be a result of blood brain barrier dysregulation and disruption of the ubiquitin proteasome system. Our findings suggest that dysregulated Keratin 9 expression is a consequence of AD pathology but, as it interacts with a broad range of proteins, it may have other, as yet uncharacterized, downstream effects which could contribute to AD onset and progression.

Keratin 9 is a 623 amino acid type 1 cytokeratin expressed predominantly in the skin^1^. Mutations within the KRT9 gene (Keratin 9) have been strongly associated with the skin disorder Epidermolytic palmoplantar keratoderma^2^,^3^,^4^. More recently however, evidence has emerged demonstrating alternative expression sites of Keratin 9 including natural killer cell membranes^5^, intestinal extracellular exosomes^6^, sperm nuclei^7^, tears^8^, follicular fluid^9^ and blood serum^10^ with several of these studies implicating the molecule in disease pathology. Dysregulated expression of Keratin 9 has, for example, been implicated in polycystic ovary syndrome^9^ and suggested as a metastatic marker of hepatocellular carcinoma^10^.

Of particular interest is recent evidence suggesting that Keratin 9 may be implicated in neurological disorders including dementia. Cerebrospinal fluid (CSF), a fluid produced by brain ventricles and which surrounds the central nervous systems, has been a target for studies into neurological disorders as it is contiguous with brain interstitial fluid and is, therefore, indicative of brain pathology^11^. A review of the CSF proteome found evidence of Keratin 9 expression in CSF using both ESI Ion Trap/FT-ICR (18 peptide sequences) and MALDI TOF/TOF (23 peptide sequences)^12^ mass spectrometry. These findings were further substantiated by a 2012 study in which 2D electrophoresis examination of CSF demonstrated that levels of Keratin 9 expression in both Multiple Sclerosis and Neuromyelitis Optica patients were twice that of control individuals^13^.

Alzheimer’s disease (AD), the most common form of dementia^14^, is posing a huge societal problem and indicators of disease that can be utilised in identification and monitoring of disease progression are vital^14^. A proteomic study of CSF undertaken by Vafadar-Isfahani *et al.* identified Keratin 9 as an important component of a biomarker panel for diagnosing AD^15^. The power of these markers resided in their combined use as a complete biomarker panel, but the existence of any marker within the panel is suggestive of some involvement in the mechanisms underlying AD pathology. A subsequent study undertaken within our laboratory utilised immunoassays to examine Keratin 9 expression in ‘matched’ CSF and blood plasma samples collected simultaneously from individual donors^16^. This study provided further validation of the presence of Keratin 9 in both CSF and blood plasma. Interestingly, Keratin 9 was detected exclusively in CSF collected from patients with AD but not in healthy individuals. Whilst the majority of the other components of the biomarker panel^15^ had previously been linked to AD^17^,^18^,^19^,^20^,^21^, it was thought that these studies were the first to implicate Keratin 9 (and also Tetranectin) in AD pathology. It has since emerged, however, that a previous study by Mueller and colleagues in 2010 used mass spectrometry to demonstrate dysregulation of Keratin 9 expression levels in AD^22^ substantiating our previous findings^15^,^16^. In addition to this, a recent study undertaken by Li *et al.* identified Keratin 9 amongst a number of potential molecules and pathways involved in AD pathology, although its involvement within these mechanisms was not expanded upon^23^.

Whilst the understandable focus of many biomarker studies, including those mentioned above, is to identify molecules with high diagnostic utility they do also offer the often overlooked opportunity to obtain a further understanding of the mechanistic origins of any disease. As evidence lends strength to the potential importance of Keratin 9 in AD, it also raises the question of how a protein typically associated with the skin can be intimately involved in a neurodegenerative disorder. The question of what has led to dysregulation of its normal expression pattern therefore requires attention and particularly whether it is a cause or consequence of the disease process.

In the present study we aim to shed light on the potential involvement of Keratin 9 in AD. Expression patterns of Keratin 9 in blood plasma samples from AD and healthy patient cohorts are examined to determine its diagnostic utility as a stand-alone biomarker and its relationship with other AD-associated protein targets. This is followed by a detailed *in silico* investigation of potential mechanisms and pathways through which Keratin 9 could influence disease pathology. These *in silico* studies were undertaken using the VisANT pathways analysis package developed at Boston University and based upon the Predictome database (http://visant.bu.edu)^24^. This software enables examination of the biological interactions of an experimentally determined biomarker, facilitating construction of molecular pathways and networks that are associated with the disease condition. In a previous study we applied VisANT analysis protocols to AD-related gene and protein panels in order to explore and redefine the mechanistic basis of the disease^25^. Other studies that utilised VisANT include investigations into the mammalian 14-3-3-phosphoproteome^26^, the involvement of the Stat5a network in prostate cancer^27^ and the role of FoxP1 in the regulation of autism-related pathways^28^. Identification of molecular pathways of which Keratin 9 is a potential component allows discussion on how Keratin 9 expression could influence or be influenced by AD pathology. Understanding the mechanisms which are associated with these pathways therefore could lead to the identification of novel drug targets or additional diagnostic biomarkers.

## Methods

### Patient samples

This study was approved by the local Ethics Committee, NRES Committee East Midlands, Ref 12/EM/0052. All methods were carried out in accordance with the approved guidelines and all participants provided written informed consent. Case samples were diagnosed as either confirmed or probable AD according to the Consortium to Establish a Registry for Alzheimer’s disease (CERAD) guidelines. All patients were diagnosed as Late-Onset Alzheimer’s disease (LOAD). Patients with significant cognitive comorbidity, including but not limited to head trauma, alcoholism, learning disability or Parkinson’s disease, were excluded from the study. The demographics of the sample cohorts analysed within this study are identified in Table 1.

### Protein detection by Immunoassay: ELISAs

Keratin 9 was detected according to the manufacturer’s protocol using an ELISA kit purchased from antibodies-online GmbH (ABIN417500). Plasma and CSF samples (diluted 1:1) were incubated in wells pre-coated with a capture antibody for 2 hr followed by incubation with a biotinylated detector antibody for 1 hr and a 30 min incubation with horseradish peroxidase-conjugated avidin. The reaction was developed using TMB substrate solution, stopped with 1 M HCl and read at 450 nm. All assay steps were undertaken at 37 °C. All samples and standard curves were measured in duplicate and concentrations of Keratin 9 in the clinical samples were determined from the standard curve generated using the standards provided in the kit.

Amyloid beta peptide 42 (Aβ42) was detected using a commercially available ELISA kit processed according to manufacturer’s instructions (Wako Chemicals GmbH; 296–64401). All samples and standard curves were measured in duplicate and concentrations of Aβ42 in the clinical samples were determined from the standard curve generated using the reagents provided in the kit.

SPARC-like 1 (SPARCL1) was measured by ELISA as previously described^16^. Briefly, ELISA plates were coated overnight with 5 μg/ml capture antibody (CAB-701MH in PBS; Creative Biomart). Following this and all subsequent incubations wells were washed three times with PBS-0.05% Tween20 (PBST). A blocking solution of PBST-1% BSA was applied to antibody-coated wells for 1 hr prior to addition of 50 μl sample (either clinical sample or protein standard) for 2 hr. Biotinylated detection antibody (BAF2728; R&D Systems) was added at 500 ng/ml for 2 hr and the reaction was developed using streptavidin-HRP and TMB substrate, stopped with 1 M HCl. Samples were read at 450 nm with concentrations of SPARCL1 determined from a standard curve generated with recombinant SPARCL1 (R&D Systems; 2728-SL).

### Protein detection by Immunoassay: Luminex assays

Apolipoprotein E (ApoE), Clusterin, Fibrinogen and Tau concentrations were determined using Luminex assays developed in-house as previously described^16^. Briefly, monoclonal capture antibodies to the target proteins were coupled to COOH-coated fluorescently dyed microspheres (Bio-Rad, Hercules, CA) using the Bio-Plex Amine Coupling Kit (Bio-Rad, 171–406001) according to manufacturer’s instructions and as previously validated^16^. A bead suspension containing 5000 of each antibody-conjugated bead set was added to designated wells of a filter plate (pre-wetted with wash buffer (PBST)), washed twice with PBST and resuspended in incubation buffer (PBS-1% BSA). To this, a sample of appropriately diluted clinical sample or protein standard mixture was added, and the plate was incubated for 2 hr at 25 °C on a rotating plate shaker (600 rpm). Wells were washed, incubated with a cocktail of biotinylated detector antibodies (containing each antibody at a pre-determined optimal concentration) for 1 hr, washed again and incubated for 30 mins with streptavidin-RPE. Wells were then washed to remove unbound streptavidin-RPE prior to analysis. Data was acquired on a Bio-Plex 200 system and analysed with associated software (Bio-Rad). All samples and standard curves were performed in duplicate and in each well, a minimum of 100 beads per target molecule were analysed for both bead designation and R-phycoerythrin fluorescence. Concentrations of the target proteins within the clinical samples were determined from standard curves generated using recombinant proteins.

### *In silico* analysis

*In silico* protein association studies were undertaken using the free licence software VisANT (http://visant.bu.edu)^24^. This network analysis tool is constructed around the Predictome database^29^ which contains details of experimentally-derived biological interactions collated from a variety of data sources^24^. An initial search of Keratin 9 interactions was undertaken to identify binding partners of the molecule. The potential contribution of Keratin 9 to AD pathology was then examined through investigation of the biological interactions of Keratin 9 with the well-established AD-associated molecules ApoE, amyloid precursor protein (APP) and Tau^30^,^31^,^32^. Searches were undertaken using the primary UniProt accession number of the appropriate proteins; P35527 (Keratin 9), P05067 (APP), P02649 (ApoE) and P10636 (Tau). An interactome was defined as the network of interactions that occurs between the target molecules under consideration. Interactions were restricted to those which occur no more than one degree away from the inputted molecules. The components identified within the derived interactome were further analysed using the free, open access pathway database Reactome (www.reactome.org)^33^ to identify potential pathways through which they may contribute to AD pathology.

### Statistics

Data were analysed using GraphPad Prism Version 6.04. Cohorts were compared using the Mann-Whitney test and Spearman correlation coefficients were determined as appropriate. A p-value ≤ 0.05 was considered to be a practical level of clinical significance. Significance values are denoted by asterisks as defined by GraphPad Prism version 6.04: ****extremely significant (p < 0.0001); ***extremely significant (0.001 ≤ p ≤ 0.0001); **very significant (0.01 ≤ p ≤ 0.001); *significant (0.05 ≤ p ≤ 0.01).

## Results & Discussion

### Keratin 9 Expression and Correlations with Other AD-associated Proteins

**Figure 1:**
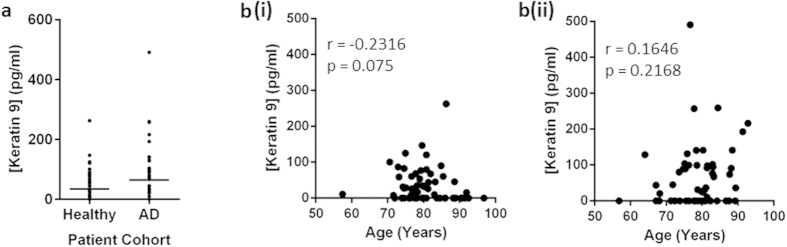
Keratin 9 expression in blood plasma in healthy and AD patient cohorts (**A**) and the correlation of these concentrations with the age of healthy (**B(i)**) and AD (**B(ii)**) individuals.

Keratin 9 expression levels were examined in blood plasma samples from a cohort comprising 60 healthy individuals and 58 AD patients, as shown in Fig. 1A. The actual concentrations observed in this cohort were lower than those obtained for an alternative cohort in a previous study^16^, however, the trends observed were analogous, with expression levels decreasing in healthy individuals (Mean = 34.7 ± 47.6 pg/ml) when compared to AD patients (Mean = 64.4 ± 88.0 pg/ml). The variation in concentrations observed between the different studies may be due to the use of different anticoagulants during blood collection, a factor which is known to affect metabolite concentrations^34^. Whilst still not reaching significance, the p-value of the change due to AD decreased for the larger cohort (p = 0.1399; n = 58 AD/60 Healthy) indicating that it may continue to improve as the sample size increases. In Fig. 1B, there appears to be some level of association between blood plasma Keratin 9 concentration and age in healthy individuals, (r = −0.2316; p = 0.075) which is dysregulated in the AD cohort (r = 0.1646; p = 0.2168).

In order to gain an understanding of the role of Keratin 9 in AD, interactions with other AD-associated proteins were sought. Immunoassays of Aβ42, ApoE, Clusterin, Fibrinogen, SPARCL1 and Tau^15^,^16^,^35^ were undertaken on blood plasma samples from the two patient cohorts. The concentrations of these proteins are detailed in Table 2. It should be noted that, whilst not the focus of this study, a significant difference (p = 0.0063) between the concentration of fibrinogen in the healthy (49.59 ± 20.72 μg/ml) and AD (41.57 ± 21.72 μg/ml) patient samples was observed lending weight to previous studies which identify it as a potential biomarker of AD^36^. Additionally, the correlations between age and concentration of ApoE, SPARCL1 and Tau were found to be disrupted upon onset of AD whilst the correlation between Aβ42 and age strengthened in the AD cohort when compared to healthy individuals (Table 2).

When the relationship between concentrations of Keratin 9 and Aβ42, ApoE, Clusterin, Fibrinogen, SPARCL1 and Tau were examined (Table 3), significant correlations were demonstrated between blood plasma concentrations of Keratin 9 and ApoE, Clusterin or Tau. The strength of all these correlations was found to increase in the AD cohort when compared to the healthy individuals. A correlation with SPARCL1 was observed, but the strength of this association remained unchanged between the two cohorts. No correlations were identified between blood plasma concentrations of Keratin 9 and Aβ42 or Fibrinogen in either the healthy or AD cohorts (Table 3).

Before speculating on the meaning of these results, the possibility of contamination was also addressed. Due to the expression of Keratin 9 in the outer, terminally differentiated epidermis, it is present in shed skin, which is a major component of dust. However unlikely, it is conceivable, that results from some previous studies could have attributed dust contamination to the detection of Keratin 9^37^. Furthermore, taking blood and CSF samples involves puncturing the skin, and as such may be a route through which keratinocyte components could enter the clinical samples. ELISA measurements of Keratin 9 in both AD patients and healthy controls collected within this study suggest that there is some level of expression in both cohorts (Fig. 1A). Whilst the possibility of contamination causing these baselines levels of expression cannot be disregarded, it cannot account for studies where differences in expression levels have been identified in identically handled samples. Work in our laboratory has previously identified the presence of Keratin 9 in AD CSF but not in normal CSF^16^ substantiating the significance of our findings and suggesting that there is a biological basis behind the results as opposed to contamination.

### Initial *in silico* Analysis

**Figure 2:**
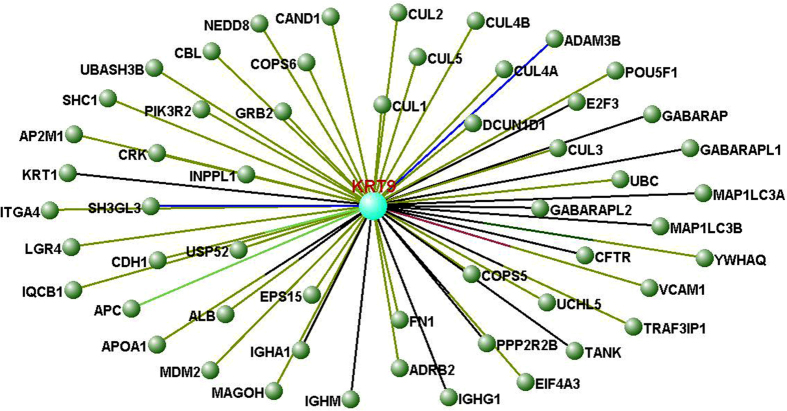
A basic Keratin 9 interactome determined using VisANT software. The interactome comprises the 54 proteins demonstrated to interact directly with Keratin 9 with the lines linking each node colour coded according to method used to establish the interaction. Further information on the nodes and interactions can be found in Table 4.

The data outlined above suggests that Keratin 9 may be implicated in the mechanistic pathways underlying AD. To further ascertain the nature of association, *in silico* protein association studies were undertaken using VisANT software, as described in Methods. Initially, the known interacting partners of Keratin 9 were examined with the interactome produced by these molecules illustrated in Fig. 2. This identified 54 proteins that have been experimentally proven to interact with Keratin 9, which are listed in Table 4, along with the experimental methods used to ascertain these interactions. Current knowledge of the function of Keratin 9 is limited; it has been shown to provide scaffolding to cells^3^ and act as an epidermal differentiation marker^38^. This VisANT analysis implies it may have further complex roles as it interacts with proteins that possess a wide range of functions (Table 4). The names and details of these proteins are listed in Table 4. Additionally, the interactome produced substantiates recent findings that Keratin 9 may have alternate expression sites to its well-characterised expression in palms and soles. For example, Keratin 9 has been shown to interact with IQCB1^39^, which is localized in cilia of renal epithelial cells and, interestingly photoreceptor cells^40^. This adds weight to the recent discovery that Keratin 9 can be found in the proteome of tears^8^. One of the most important implications of this interactome is that it provides further validation of the presence of Keratin 9 in blood plasma^10^,^16^ as it can interact with five of the most abundant proteins in blood serum: Albumin, Apolipoprotein A-I and the heavy chain constant regions of Immunoglobins A, G and M^41^.

### Further *in silico* Analysis: Expanding the Keratin 9 Interactome

**Figure 3:**
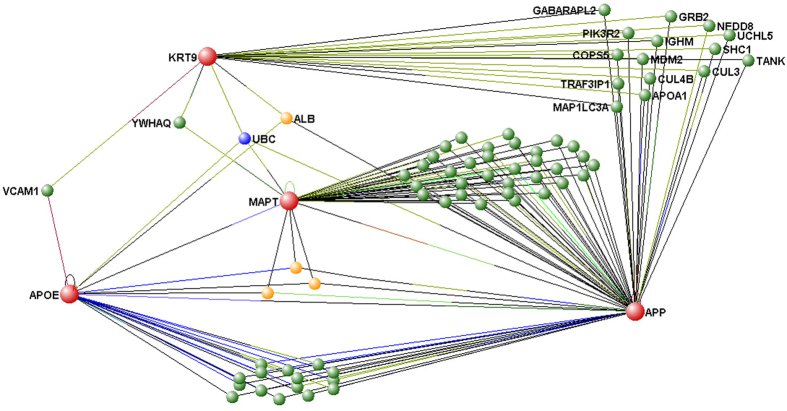
The Interactome of Keratin 9, Apolipoprotein E (APOE), Tau (MAPT) and Amyloid Precursor Protein (APP) (nodes shown in red). VisANT software was used to identify all interacting molecules. Interacting molecules are identified as green (interacting with 2 proteins), yellow (interacting with 3 proteins), or blue (interacting with 4 proteins). Proteins with only a single interaction were excluded from the Interactome. Only the nodes with direct links to Keratin 9 have been labelled.

Although this interactome provides a useful start point from which to understand the involvement of Keratin 9 in AD, it only details direct molecular interactions; expanding the interactome would highlight further biochemical pathways that may influence/be influenced by variations in Keratin 9 expression. With this in mind, we examined the additional interactions of all 54 identified Keratin 9 interacting proteins. However, simply expanding every node resulted in high levels of complexity in the outputted data. We proceeded, therefore, to examine the interactions between Keratin 9 and other molecules known to be involved in AD pathology directly rather than indirectly. VisANT was utilised to produce an interactome detailing the interactions of Keratin 9 with APP, Tau (MAPT) and ApoE, three molecules with a longstanding association with AD (together, referred to as the AMA interactome). Molecules found to have two or more interacting partners within the derived network (i.e. they interact with at least two out of ApoE, APP, Keratin 9 and Tau) are included in the resultant interactome (Fig. 3). Correlations between expression levels of Keratin 9 and those of ApoE and Tau were earlier found to be disrupted in blood plasma samples from AD patients (Table 3). Whilst Keratin 9 has not yet been demonstrated to interact directly with APP, ApoE or Tau, Fig. 3 demonstrates that it is closely connected to all three molecules, requiring only one bridging molecule. The molecules found to interact with Keratin 9 and the AMA interactome are summarised in Table 4. In the case of ApoE Keratin 9 is linked via either VCAM1, ALB or UBC whilst for Tau it is linked via UBC and YWHAQ and for APP, it is linked via ALB, APOA1, COPS5, CUL3, CUL4B, GABARAPL2, GRB2, IGHM, MAP1LC3A, MDM2, NEDD8, PIK3R2, SHC1, TANK, TRAF3IP1, UBC and UCHL5. These molecular associations fail, however, to shed much more light on the problem since it is unlikely that Keratin 9 levels would affect APOE, MAPT or APP and vice versa through such a short pathway. Furthermore, many of the linking molecules, such as UBC, are ubiquitously expressed and interact with a broad range of proteins so any effect manifesting through these molecules would not explain the specificity of AD symptoms. It is therefore necessary to expand the interactome network: a series of several, more specific interactions that link these proteins is potentially the key to solving the problem as opposed to searching for the shortest connection paths. One way to solve this problem, whilst avoiding generating too much complexity in the interactome, is to place Keratin 9 in the context of pathways that are already thought to play a role in AD.

In previous work we have used Reactome (http://www.reactome.org/)^25^,^42^ to identify pathways in which AD biomarkers may be participating^25^. Initially, the same approach was implemented here whereby Keratin 9 and all 54 of its interacting molecules that were identified through VisANT were inputted into Reactome. However 10 out of these 55 molecules, including Keratin 9, were not found in the Reactome database, and as a consequence this method was deemed inappropriate for determining the pathways associated with Keratin 9. Instead, the functions of each of the Keratin 9 interacting proteins were evaluated using the UniProtKB database, which displays the complete Gene Ontology annotation as well as a full list of Reactome entries for each protein. Additionally, since VisANT only displays proteins that share physical interactions, we searched the literature for evidence of transcriptional regulation of Keratin 9 to establish whether Keratin 9 regulators play a role in AD. As a result of this, several pathways linked to these interacting proteins and regulatory proteins were highlighted, including wnt signalling and the ubiquitin proteasome system which have already been linked to AD^43^,^44^,^45^.

### Wnt Signalling in AD

The canonical wnt signalling pathway regulates the expression of a wide range of genes throughout the body and has been strongly implicated in AD^43^,^44^. The cascade is initiated when wnt ligands complex with LRP5/6 and Frizzled (Fz) receptors. Fz then activates Dishevelled protein, which forms a complex with GSK3β, adenomatous polyposis coli (APC), Axin and beta-catenin resulting in inactivation of GSK3β and stabilization of beta-catenin. Beta catenin can then bind to T cell receptor (Tcf)/Lymphoid enhancer-binding factor (LEF) transcription factors by displacement of the repressor protein, Groucho leading to activation or repression of target genes. In the absence of wnt, or in the presence of a wnt antagonist, GSK3β remains active, facilitating phosphorylation of beta-catenin, which is targeted to the ubiquitin-proteasome degradation pathway^44^. This prevents beta-catenin from entering the nucleus and interacting with transcription factors. It is thought that downregulation of components of this cascade can result in many of the key changes that occur in the AD brain: repression of wnt signalling has so far been linked to the processing of APP, Aβ peptide neurotoxicity and tau phosphorylation^43^. For example beta secretase (BACE1), the APP cleaving enzyme, is suppressed following wnt activation via beta-catenin binding to TCF4, a BACE1 promoter^46^. Therefore, downregulation of the wnt pathway can result in increased cleavage of APP and therefore increased Aβ levels.

VisANT analysis suggests that Keratin 9 may be linked to this cascade via interactions with APC and Cadherin 1 (CDH1), which interacts with β-catenin in adherens junctions. It is unlikely however, that Keratin 9 could affect this pathway through binding to CDH1 or APC as there is no evidence of Keratin 9 acting as a signalling molecule. It is more likely that Keratin 9 levels could be influenced by this pathway as it has been suggested that wnt signalling may be involved in keratinocyte development. Dickkopf 1 (DKK1), an antagonist of the wnt pathway, has been shown to induce the expression of Keratin 9, resulting in a thicker epidermis^47^. Furthermore, increased expression of DKK1 has been seen in brain tissue of AD patients and this has been causally linked to the neurodegeneration associated with the disease^48^. It is thought that expression of Aβ induces expression of DKK1 in a P53 dependent manner and conversely, repressing DKK1 reduces Aβ toxicity^49^. Increased expression of DKK1 could therefore not only help explain the characteristic amyloid beta toxicity, but also the presence of Keratin 9 in the CSF of AD patients. Although, to our knowledge, this is the first time a link has been proposed between DKK1 and Keratin 9 in non-epidermal tissue, both DKK1 and Keratin 9 have separately been shown to have diagnostic potential for hepatocellular carcinoma due to their dysregulation in blood serum^10^,^50^. This suggests that, in certain disease conditions, DKK1 could induce the expression of Keratin 9 in non-epidermal tissue.

Interestingly, our results also show a correlation between changes in Keratin 9 and Clusterin levels in AD (Table 3). This could be explained by previous research by Killick and colleagues^49^, which suggests linkages between Clusterin, DKK1 and Aβ. They found that knockdown of Clusterin expression results in reduced Aβ toxicity and DKK1 expression, suggesting that Aβ induced DKK1 expression is dependent on Clusterin, as well as P53. They also highlighted a potential mechanism, thought to be the amyloid cascade, whereby Clusterin mediates neurotoxicity in an Aβ induced clusterin/P53/DKK1/wnt-PCP-JNK pathway. Since our immunoassay detection did not include DKK1 or P53, we can only speculate that these may be linked to Clusterin and Keratin 9 based on previous research. It would therefore be of interest to include DKK1 and P53 in our biomarker panel in future in order to establish for ourselves whether these correlate with Clusterin, Aβ or Keratin 9.

### Keratin 9 and Blood Brain Barrier Dysregulation in AD

The presence of Keratin 9 in CSF has previously been demonstrated using various mass spectrometry techniques^12^,^15^ and 2D electrophoresis^13^, but these techniques gave no indication of the concentration at which it existed. To our knowledge, the first immunoassay validation of the presence of Keratin 9 in CSF was obtained during our previous study which demonstrated the presence of Keratin 9 in the CSF of AD patients but not in healthy individuals^16^. Keratin 9 has also been identified in the proteome of blood plasma multiple times^41^,^51^,^52^,^53^,^54^,^55^,^56^,^57^,^58^,^59^,^60^,^61^,^62^. Although sample contamination cannot be ruled out, and we can only speculate on why Keratin 9 is present in blood, the sheer number of studies documenting this result adds plausibility to our finding of Keratin 9 in blood plasma in both healthy and AD patients. Furthermore, our finding that Keratin 9 was present in the CSF of AD individuals but not in healthy individuals^16^, could suggest that there are differences in the blood brain barrier (BBB), enabling Keratin 9 to pass from the blood into CSF in AD individuals.

Dysfunction of the BBB has already been strongly implicated in AD. The integrity of the BBB is compromised in AD due to down-regulation of Claudin proteins, resulting in destabilisation of tight junctions between endothelial cells^63^. Evidence also suggests that p-glycoprotein, an Aβ transporter at the BBB, is down-regulated in AD, resulting in compromised Aβ clearance and accumulation of Aβ in the brain^64^. Interestingly, expression of p-glycoprotein and maintenance of tight junctions in the BBB are known to be regulated by the wnt signalling pathway^63^,^65^. Increased expression of DKK1, as mentioned previously, inhibits this pathway and therefore contributes to increased BBB permeability, decreased p-glycoprotein expression and Aβ accumulation. Increased permeability of the BBB could therefore enable Keratin 9, which is up-regulated by DKK1^47^, to pass from blood to CSF in AD patients.

### Keratin 9 and the Ubiquitin Proteasome System in AD

The interactome produced using VisANT and subsequent analysis revealed that at least 12 of the Keratin 9 interacting partners: CAND1, CBL, CUL1, CUL2, CUL3, CUL4A, CUL4B, CUL5, DCUN1D, MDM2, NEDD8 and UBC, are components of the Ubiquitin Proteasome System (UPS)^66^,^67^,^68^,^69^. This suggests that Keratin 9 may be ubiquitinated and targeted for degradation by the proteasome. However, the abnormal presence of Keratin 9 in CSF and the elevated levels of Keratin 9 in blood of AD patients indicate a failure in this system, which prevents Keratin 9 degradation. Dysregulation of the UPS has previously been linked to AD as a significant decrease in proteasome activity has been observed in various regions of the AD brain and this is thought to result in the aggregation of Aβ into plaques^45^. This may also explain the build-up of Keratin 9 and therefore Keratin 9 may be a useful biomarker to detect the UPS dysregulation associated with AD.

### Keratin 9: More than just a structural molecule

In addition to functioning as scaffolding proteins within cellular cytoskeletons, some keratins are known to be involved in cell signalling, communicating with extracellular matrix (ECM) components and other cells through desmosomes and hemidesmosomes^70^,^71^. Although Keratin 9 has not yet been implicated in any signalling pathways, VisANT analysis reveals interactions with several cytoskeletal and ECM components, implying a potential signalling role. Specifically, Keratin 9 interacts with CDH1 and ITGA4, which are involved in cell-cell junctions and cell-matrix interactions, and FN1, a component of the ECM. Keratin 9 also interacts directly with other cytoskeletal components, including KRT1 and indirectly with microtubules through interactions with MAP1LC3A and MAP1LC3B. There is a wide range of other proteins with which Keratin 9 also interacts (Table 4) for which the implications of the interactions are currently unclear. As these proteins have a variety of functions it could be speculated that the interactions may have unforeseen consequences for other downstream pathways. If this were to be proven and if the affected pathways influence AD it would mean that dysregulation of Keratin 9 expression, which currently appears to be a consequence of AD, may have consequences of its own that may contribute to AD onset and progression.

## Conclusion

**Figure 4:**
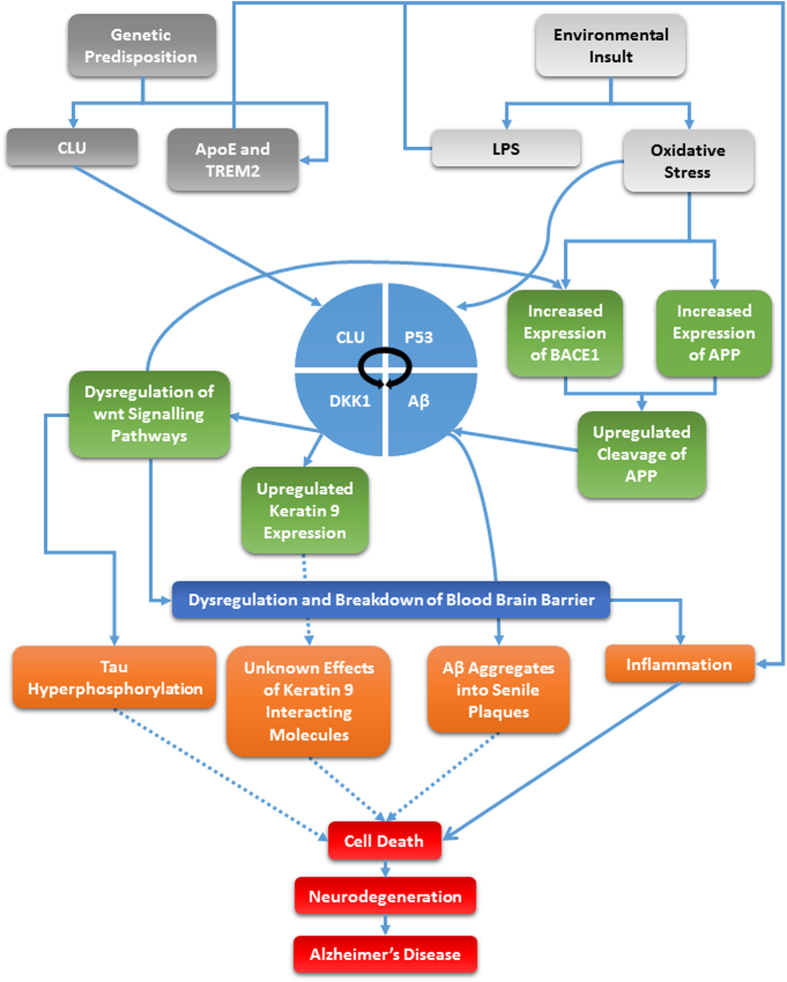
Keratin 9 in Alzheimer’s disease pathology. The diagram illustrates how Keratin 9 may interact with some of the major known factors in Alzheimer’s disease including those pathways which implicate ApoE, APP, Clusterin and Tau. Dotted lines indicate unknown mechanisms.

Rapidly emerging evidence suggests that Alzheimer’s disease is multifactorial and therefore, finding a single marker for the disease is not the key to early diagnosis. With this in mind, we do not present Keratin 9 as a stand-alone biomarker for the disease but rather, a useful component of a biomarker panel. The use of Keratin 9 as a biomarker seems controversial and counterintuitive considering that is was until recently believed to be solely expressed in terminally differentiated layers of the epidermis. However, biochemical analysis of expression patterns and *in silico* mapping of keratin 9 interactions has proved to be useful in understanding potential mechanisms that may explain the involvement of keratin 9 in the disease. These pathways, alongside other proposed AD risk factors and their likely role in AD pathology are summarized in Fig. 4. We can speculate from our research that the abnormal expression of keratin 9 in AD is likely the consequence of dysregulated signalling pathways, notably wnt signalling pathways and potentially the UPS, in addition to a break-down in the BBB. Based on this information, we suggest that P53 and DKK1 should also be considered as potential biomarkers in addition to the panel of markers we identified previously. Furthermore, the interactome of keratin 9 also suggests that its abnormal expression may have other downstream consequences resulting from its interactions with a broad range of other molecules, so this may be a potential area for future research.

## Additional Information

**How to cite this article**: Richens, J. L. *et al.* Rationalising the role of Keratin 9 as a biomarker for Alzheimer’s disease. *Sci. Rep.* **6**, 22962; doi: 10.1038/srep22962 (2016).

## Figures and Tables

**Table 1:** Patient demographics of the blood plasma samples used within this study.

	Healthy Cohort	AD Cohort
Sample number	60	58
Gender (M/F)	25/35	31/27
Age at entry (years ± SD)	80.33 ± 6.64	79.03 ± 6.96
Time between study entry and recalled onset (years ± SD)	—	4.32 ± 2.71

**Table 2:** Blood plasma concentrations of putative AD biomarker targets as measured by immunoassay.

Molecule	Healthy Cohort			AD Cohort		
	Concentration	Correlation with age		Concentration	Correlation with age	
		Correlation coefficient (r)	p-value		Correlation coefficient (r)	p-value
Aβ42	6.49 ± 1.25 pmol/L	0.0755	0.6432	6.32 ± 1.74 pmol/L	0.3802	0.0185*
ApoE	4776 ± 3238 ng/ml	−0.3833	0.0025**	4607 ± 2689 ng/ml	0.1369	0.3056
Clusterin	2534 ± 1397 ng/ml	−0.2536	0.0505	2516 ± 1336 ng/ml	0.0824	0.5386
Fibrinogen	49.59 ± 20.72 μg/ml	0.0570	0.6652	41.57 ± 21.72 μg/ml	0.2234	0.0918
Keratin 9	34.7 ± 47.6 pg/ml	−0.2316	0.0750	64.4 ± 88.0 pg/ml	0.1646	0.2168
SPARCL1	1126 ± 533 ng/ml	0.4619	0.0002***	1204 ± 757 ng/ml	0.1711	0.1992
Tau	43.90 ± 13.78 ng/ml	−0.4082	0.0012**	43.10 ± 14.84 ng/ml	0.1062	0.4275

Spearman correlation coefficients for protein concentration and patient age were determined using GraphPad Prism Version 5.02. A *p*-value ≤ 0.05 was considered statistically significant.

**Table 3:** Correlations between protein concentrations of Keratin 9 and other AD-associated molecules in blood plasma samples.

Molecule 1	Molecule 2	Healthy Cohort		AD Cohort	
		Spearman correlation coefficient (r)	p-value	Spearman correlation coefficient (r)	p-value
Keratin 9	Aβ42	0.1045	p = 0.5209	−0.05246	p = 0.7478
Keratin 9	ApoE	0.3829	p = 0.0025**	0.6380	p < 0.0001****
Keratin 9	Clusterin	0.2707	p = 0.0364*	0.5276	p < 0.0001****
Keratin 9	Fibrinogen	0.1107	p = 0.3998	0.03512	p = 0.7899
Keratin 9	SPARCL1	−0.3026	p = 0.0188*	−0.3102	p = 0.0158*
Keratin 9	Tau	0.4338	p = 0.0005***	0.6256	p < 0.0001****

Protein concentrations were determined using the appropriate ELISA of Luminex assay. Spearman correlation coefficients were determined for all protein pairings in healthy and AD patient cohorts using GraphPad Prism Version 5.02. A *p*-value ≤ 0.05 was considered statistically significant.

**Table 4:** Components of the Keratin 9 interactome determined using VisANT software.

KRT9 interacting partner	Experimental Evidence of Interaction	Reference	Direct Link to AMA Interactome		
			APP	APOE	MAPT
ADAM metallopeptidase domain 3B (ADAM3B)	Two-hybrid Test	72	—	—	—
Adrenoceptor beta 2, surface (ADRB2)	Affinity Capture-MS	73	—	—	—
Albumin (ALB)	Affinity Capture-MS; Anti-Bait Coimmunoprecipitation	41	✓	✓	—
Adaptor-related protein complex 2, mu 1 subunit (AP2M1)	Affinity Capture-MS	67	—	—	—
Adenomatous polyposis coli (APC)	Surface Plasmon Resonance	74	—	—	—
Apolipoprotein A-I (APOA1)	Affinity Capture-MS; Anti-Bait Coimmunoprecipitation	41	✓	—	—
Cullin-associated and neddylation-dissociated 1 (CAND1)	Affinity Capture-MS	66	—	—	—
Cbl proto-oncogene, E3 ubiquitin protein ligase (CBL)	Affinity Capture-MS	67	—	—	—
Cadherin 1, type 1, E-cadherin (epithelial) (CDH1)	Affinity Capture-MS; Surface Plasmon Resonance	74	—	—	—
Cystic fibrosis transmembrane conductance regulator (CFTR)	Anti-Bait Coimmunoprecipitation	75	—	—	—
COP9 signalosome subunit 5 (COPS5)	Affinity Capture-MS	66	✓	—	—
COP9 signalosome subunit 6 (COPS6)	Affinity Capture-MS	66	—	—	—
V-crk avian sarcoma virus CT10 oncogene homolog (CRK)	Affinity Capture-MS	67	—	—	—
Cullin 1 (CUL1)	Affinity Capture-MS	66	—	—	—
Cullin 2 (CUL2)	Affinity Capture-MS	66	—	—	—
Cullin 3 (CUL3)	Affinity Capture-MS	66	✓	—	—
Cullin 4A (CUL4A)	Affinity Capture-MS	66	—	—	—
Cullin 4B (CUL4B)	Affinity Capture-MS	66	✓	—	—
Cullin 5 (CUL5)	Affinity Capture-MS	66	—	—	—
DCN1, defective in cullin neddylation 1, domain containing 1 (DCUN1D)	Affinity Capture-MS	66	—	—	—
E2F transcription factor 3 (E2F3)	Pull Down	76	—	—	—
Eukaryotic translation initiation factor 4A3 (EIF4A3)	Affinity Capture-MS	77	—	—	—
Epidermal growth factor receptor pathway substrate 15 (EPS15)	Affinity Capture-MS; Anti-Tag Coimmunoprecipitation	67	—	—	—
Fibronectin 1 (FN1)	Affinity Capture-MS	78	—	—	—
GABA(A) receptor-associated protein (GABARAP)	Anti-Tag Coimmunoprecipitation	79	—	—	—
GABA(A) receptor-associated protein like 1 (GABARAPL1)	Anti-Tag Coimmunoprecipitation	79	—	—	—
GABA(A) receptor-associated protein like 2 (GABARAPL2)	Anti-Tag Coimmunoprecipitation	79	✓	—	—
Growth factor receptor-bound protein 2 (GRB2)	Affinity Capture-MS	67	✓	—	—
Immunoglobulin heavy constant alpha 1 (IGHA1)	Anti-Bait Coimmunoprecipitation	41	—	—	—
Immunoglobulin heavy constant gamma 1 (IGHG1)	Anti-Bait Coimmunoprecipitation	41	—	—	—
Immunoglobulin heavy constant mu (IGHM)	Anti-Bait Coimmunoprecipitation	41	✓	—	—
Inositol polyphosphate phosphatase-like 1 (INPPL1)	Affinity Capture-MS	67	—	—	—
IQ motif containing B1 (IQCB1)	Affinity Capture-MS	39	—	—	—
Integrin, alpha 4 (ITGA4)	Affinity Capture-MS	80	—	—	—
Keratin 1 (KRT1)	Co-Fractionation	81	—	—	—
Leucine-rich repeat containing G protein-coupled receptor 4 (LGR4)	Affinity Capture-MS	82	—	—	—
Mago-nashi homolog, proliferation-associated (MAGOH)	Affinity Capture-MS	77	—	—	—
Microtubule-associated protein 1 light chain 3 alpha (MAP1LC3A)	Anti-Tag Coimmunoprecipitation	79	✓	—	—
Microtubule-associated protein 1 light chain 3 beta (MAP1LC3B)	Anti-Tag Coimmunoprecipitation	79	—	—	—
MDM2 proto-oncogene, E3 ubiquitin protein ligase (MDM2)	Affinity Capture-MS	69	✓	—	—
Neural precursor cell expressed, developmentally down-regulated 8 (NEDD8)	Affinity Capture-MS	66	✓	—	—
Phosphoinositide-3-kinase, regulatory subunit 2 (beta) (PIK3R2)	Affinity Capture-MS	67	✓	—	—
POU class 5 homeobox 1 (POU5F1)	Affinity Capture-MS	83	—	—	—
Protein phosphatase 2, regulatory subunit B, beta (PPP2R2B)	Pull Down; Anti-Tag Coimmunopreciptation	84	—	—	—
SH3-domain GRB2-like 3 (SH3GL3)	Two Hybrid Test	72	—	—	—
Src homology 2 domain containing transforming protein 1 (SHC1)	Affinity Capture-MS	67	✓	—	—
TRAF family member-associated NFKB activator (TANK)	Functional Linkage Network; Anti-Tag Coimmunoprecipitation	85	✓	—	—
TNF receptor-associated factor 3 interacting protein 1 (TRAF3IP1)	Anti-Bait Coimmunoprecipitation; Affinity Capture-MS	86	✓	—	—
Ubiquitin associated and SH3 domain containing B (UBASH3B)	Affinity Capture-MS	67	—	—	—
Ubiquitin C (UBC)	Affinity Capture-MS	68	✓	✓	✓
Ubiquitin carboxyl-terminal hydrolase L5 (UCHL5)	Affinity Capture-MS	87	✓	—	—
PAN2 poly (A) specific ribonuclease subunit (USP52)	Affinity Capture-MS	88	—	—	—
Vascular cell adhesion molecule 1 (VCAM1)	Cross-linking Studies; Affinity Capture-MS	78,80	—	✓	—
Tyrosine 3-monooxygenase/tryptophan 5-monooxygenase activation protein, theta (YWHAQ)	*In vivo*; Coimmunoprecipitation; Affinity Capture-MS	89	—	—	✓

The means of determining their interaction with Keratin 9 and their links to the AMA Interactome are displayed.
